# A Treatise on Epidemic Cholera

**Published:** 1856-01

**Authors:** 


*A Treatise on Epidemic Cholera.* By Horatio Gates Jameson, Sr., M. D. Phila—Lindsay & Blakison, 1855.

This work has been received from the publishers. We have not time yet to read it. It seems to be very thorough, and will be noticed hereafter more fully.

